# miR-96-5p promotes the proliferation and migration of ovarian cancer cells by suppressing Caveolae1

**DOI:** 10.1186/s13048-019-0533-1

**Published:** 2019-06-22

**Authors:** Bo Liu, Jinglu Zhang, Dongxia Yang

**Affiliations:** Department of gynaecology & obstetrics, Jinan Maternal & Children Health Care Hospital, No 2 Jianguo Xiaojing Three road, Jinan, 250001 Shandong People’s Republic of China

**Keywords:** Ovarian cancer, miR-96-5p, Caveolae1, AKT signaling pathway, Proliferation, Migration

## Abstract

**Background:**

Ovarian cancer (OC) is the second most common gynaecological malignancy. MicroRNAs (miRNAs) have been found to be aberrantly expressed in OC tissue and have been proposed as biomarkers and therapeutic targets for OC.

**Results:**

In this study, we found that miR-96-5p was up-regulated in OC tissues and OC cells compared to normal ovarian tissues and epithelial cell line. And, miR-96-5p was also up-regulated in the serum samples from OC patients compared to health participants. In addition, there was a positive correlation of miR-96-5p levels between OC tissues and serum samples. At the cellular level, overexpression of miR-96-5p promoted cell proliferation and migration in OC cells. Moreover, we further validated Caveolae1 (CAV1) as the direct target of miR-96-5p in OC cells through luciferase activity assays and western blot. CAV1 was obvious low expression in OC tissues. The overexpression of CAV1 abrogated the promotion of miR-96-5p on the OC cells proliferation and migration. Finally, we found that AKT signaling pathway was involved in this process. MiR-96-5p inhibited the phosphorylation of AKT and expression of down-stream proteins Cyclin D1 and P70 by targeting CAV1.

**Conclusions:**

The above findings suggested that targeting miR-96-5p may be a promising strategy for OC treatment.

## Background

Ovarian cancer (OC) is the second most common gynaecological malignancy, accounting for approximately 4% of all females cancers in worldwide [1]. OC patients are usually diagnosed at an advanced stage due to its insidious onset. In this case, the initial treatment may cause a complete response, but these cancers often relapse. The response rates of relapsed patients are lower than 15–20% with standard treatments [1]. In addition, to date, there is no effective screening tool for early OC [1]. Therefore, this is a growing interest in the potential utility of microRNAs (miRNAs) in the treatment of OC.

MiRNAs, a class of small endogenous non-coding RNAs, could suppress the expression of target genes through base pairing to the specific sequences in the 3′ UTR of their mRNA leading to mRNA degradation or translational inhibition [2, 3]. The dyregulation of miRNAs has been observed in various types of human cancers [4, 5]. Emerging studies have suggested that miRNAs act as oncogenes or tumor suppressors, and play important roles in cancer development and progression [6, 7].

Among miRNAs, microRNA-96 (miR-96-5p) is one member of the miR-183-96-182 cluster which serves an important role in the regulation of the cancer cells’ biological behavior. MiR-96-5p has been found to be highly up-regulated in different kind of tumors, including breast cancer [8], thyroid cancer [9], bladder cancer [10], adrenocortical and adrenal medullary tumors [11], head and neck squamous cell carcinoma, and be significant down-regulated in osteosarcoma [12]. MiR-96-5p has been demonstrated to be related with the development and progression of these cancers. However, to the best of our knowledge, the relationship between miR-96-5p and OC has not been investigated so far.

Caveolae are 50- to 100-, omega-shaped invaginations of the plasma membrane [13, 14], whose roles have expanded to macromolecular vesicles transportion [14], signal transduction [15], cellular metabolism [16], cholesterol homeostasis, endocytosis, tumor promotion and tumor suppression [17]. Caveolae1 (CAV1), a 21–24 kDa protein, acts as a principal resident structural protein component of caveolae, which participates in vesicular trafficking and signal transduction through interactions with various protein and nonprotein molecules [13, 14]. Furthermore, CAV1 plays important roles in tumorigenesis and shows to act as a tumor-promoter or suppresser depending on the tumor cell types and subtypes [18, 19]. Recent researches indicate that CAV1 functions as a tumor suppressor in OC [20, 21].

AKT, an important downstream molecule of PI3K, plays an important role in regulating cell growth, proliferation, migration, survival and glucose metabolism [22]. Activated Akt directly phosphorylates PRAS40, which disables mTORC1 inhibition, thereby activating the mTORC1 pathway [23]. In regulating protein synthesis, the downstream effectors of mTORC are mainly ribosomal p70S6 kinase protein (P70) and eukaryotic initiation factor 4E binding protein 1 (4E-BP1) [24]. Activated mTORC1 phosphorylates and activates P70, which in turn phosphorylates and actives the ribosomal 40S protein S6, which ultimately initiates translation of the 5′ end of the mRNA and the encoding of ribosomal proteins and elongation factors that stimulate protein synthesis [25]. In addition, mTORC1 activates and phosphorylates 4E-BP1, participates in the formation of the eIF4F complex, initiates translation and encodes a cell cycle regulatory protein, including Cyclin D1 [24].

In this study, we provided insights into the oncogene function of miR-96-5p and network of miR-96-5p, CAV1 and AKT signaling pathway in OC.

## Results

### MiR-96-5p was up-regulated in OC tissues and cells

**Fig. 1:**
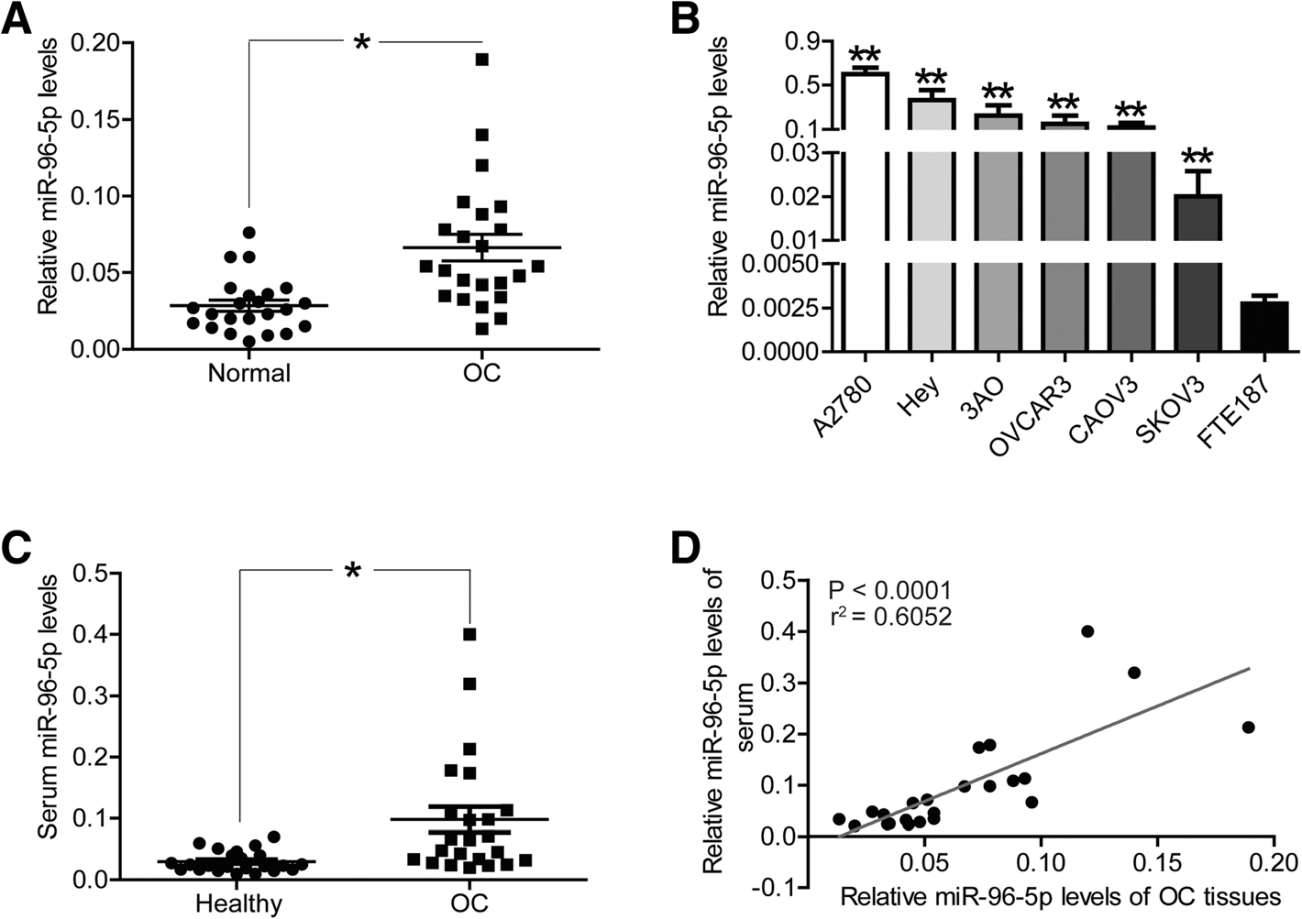
MiR-96-5p was up-regulated in OC tissues and cells, and serum samples of OC patients. **a** miR-96-5p levels in OC tissues and adjacent normal ovarian tissues (*n* = 23) were evaluated via qRT-PCR. **b** miR-96-5p levels in OC cell lines (A2780, Hey, 3AO, OVCAR3, CAOV3 and SKOV3) and immortalized normal human fallopain tube epithelial cell line (FTE187) were determined via qRT-PCR. **c** the abundance of miR-96-5p in serum samples of OC patients and health participants were detected by qRT-PCR. **d** the correlation of miR-96-5p levels between OC tissues and serum samples were determined by Pearson analysis. *P < 0.05, **P < 0.01. Normal, adjacent normal ovarian tissues; OC, OC tissues or serum samples from OC patients; Healthy, serum samples from health participants

First of all, we collected a panel OC patients (*n* = 23) and used qRT-PCR assays to confirm the expression level of miR-96-5p between OC tissues and adjacent normal ovarian tissues. The results displayed in Fig. 1a, the abundance of miR-96-5p was significantly elevated in OC tissues, in comparison with adjacent normal ovarian tissues (*P* < 0.05). Similarly, the expression level of miR-96-5p was also obviously higher in OC cell lines (A2780, Hey, 3AO, OVCAR3, CAOV3 and SKOV3) than in immortalized normal human fallopain tube epithelial cell line (FTE187) (Fig. 1b, *P* < 0.01). Considering the lower expression of miR-96-5p in SKOV3 and CAOV3 cells compared to other OC cell lines, it was selected for subsequent experiments.

### MiR-96-5p level was significantly higher in the serum samples of OC patients

Secondly, we evaluated the expression level of miR-96-5p in serum samples from OC patients or health participants. Similar to its expression profile in OC tissues, miR-96-5p was also up-regulated in the serum samples from OC patients, in comparison with health participants (Fig. 1c, *P* < 0.05). In addition, we compared the expression level of miR-96-5p between OC tissues and serum samples, and found a positive correlation of miR-96-5p between OC tissues and serum samples (Fig. 1d, *P* < 0.0001). miR-96-5p.

### The overexpression of miR-96-5p improved the malignant phenotypes of OC cells

**Fig. 2:**
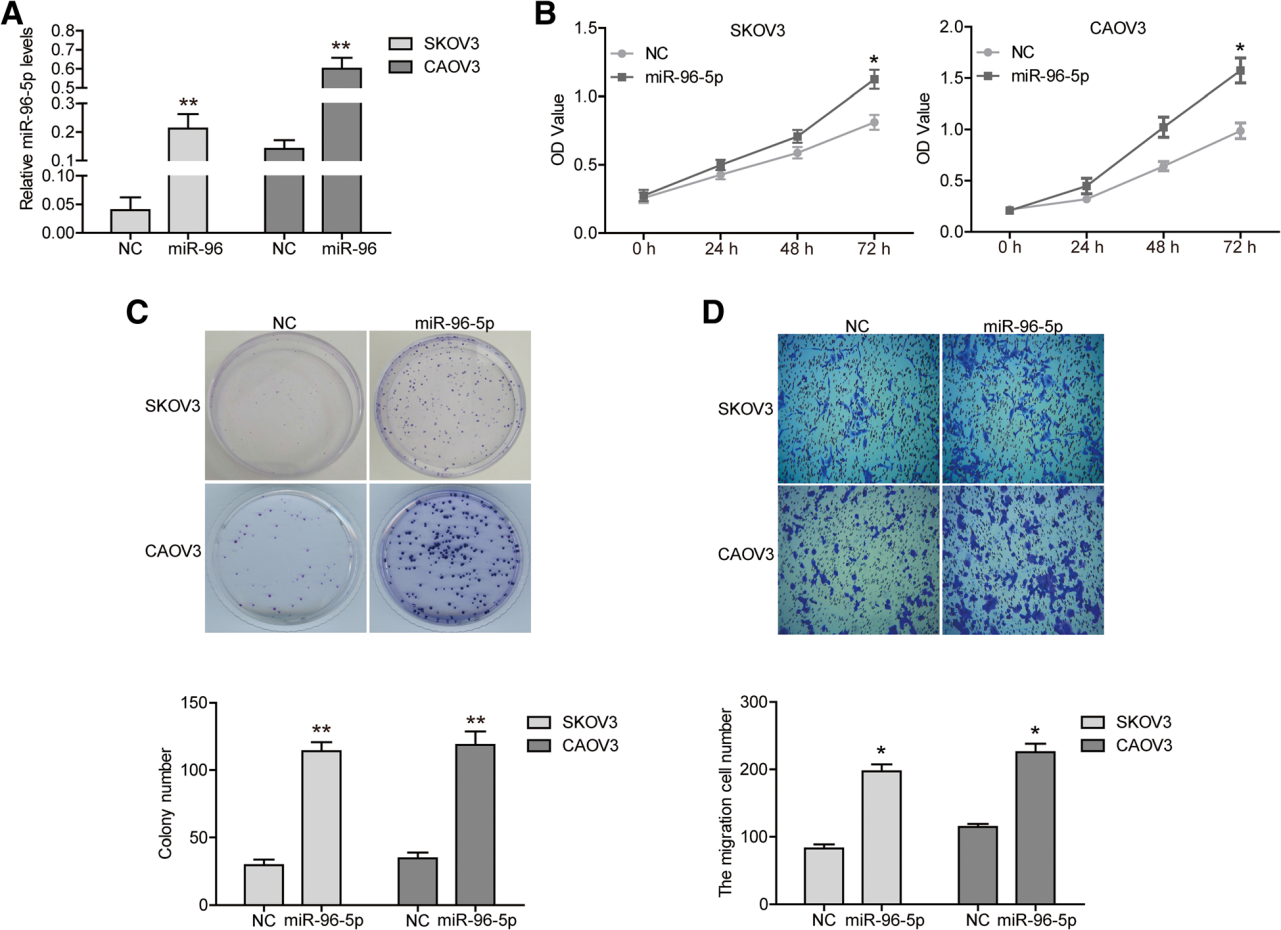
The overexpression of miR-96-5p improved the malignant phenotypes of SKOV3 cells. **a** miR-96-5p levels were evaluated via qRT-PCR. **b** the proliferation of SKOV3 and CAOV3 cells were determined via CCK8 assay. **c** the clonogenicity of SKOV3 and CAOV3 cells were detected by clony formation assay. **d** the migration of SKOV3 and CAOV3 cells were determined by transwell assay. *P < 0.05, **P < 0.01. NC, negative control; miR-96-5p, miR-96-5p mimics transfected cells

Next, we aimed to study if the change in the levels of miR-96-5p was able to affect the proliferation, clonogenicity, migration and cell cycle of OC cells. We employed miR-96-5p mimics to achieve the overexpression of miR-96-5p in SKOV3 and CAOV3 cells. And qRT-PCR assays showed that miR-96-5p levels were significantly up-regulated in both cells transfected with miR-96-5p mimics (Fig. 2a, *P* < 0.01). CCK8 assay displayed a better cell viability in miR-96-5p over-expressed cells, which meant that miR-96-5p improved cell proliferation (Fig. 2b, *P* < 0.05). By colony formation assay, cell colonies in miR-96-5p over-expressed group were enormously larger and more than those in NC group (Fig. 2c, *P* < 0.01). In addition, transwell assay results revealed more migratory cells in miR-96-5p over-expressed group than in NC group (Fig. 2d, *P* < 0.05).

**Fig. 3:**
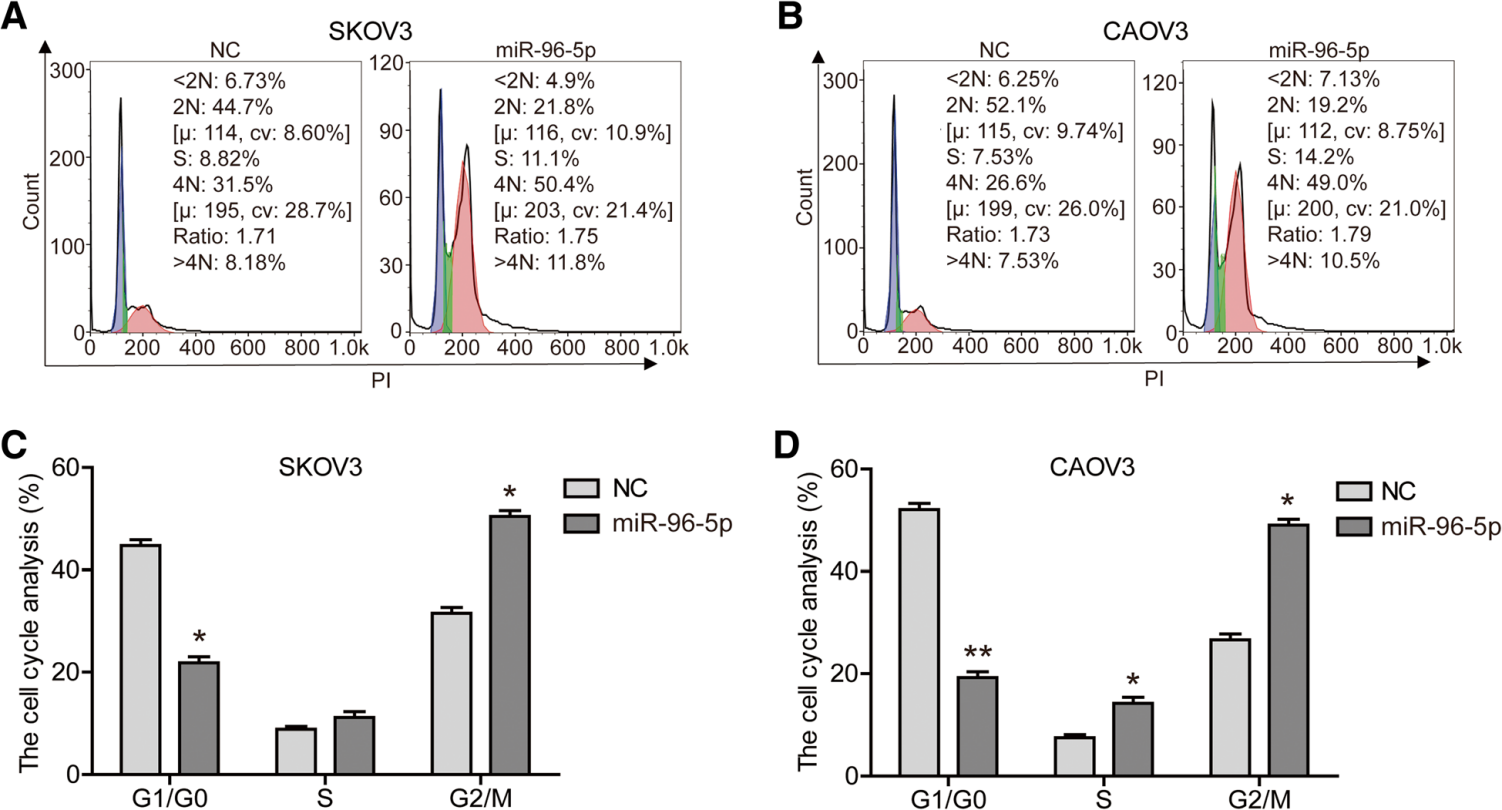
Overexpression of miR-96-5p drived the progression of cell cycle. Cell cycle of SKOV3 (**a**) and CAOV3 (**b**) cells were detected by flow cytometry. The proportion of SKOV3 (**c**) and CAOV3 (**d**) cells in each period was quantified. *P < 0.05, **P < 0.01. NC, negative control; miR-96-5p, miR-96-5p mimics transfected cells

Furthermore, to explore how miR-96-5p affected OC cell proliferation and growth, we examined the effect of miR-96-5p expression on cell cycle using flow cytometry. As shown in Fig. 3, after transfected with miR-96-5p mimic, the proportion of cells in the G1/G0 phase was significantly decreased (*P* < 0.05), while the proportion of cells in the G2/M phase was obviously increased (*P* < 0.05). This data indicated that overexpression of miR-96-5p drived the progression of cell cycle. In a word, miR-96-5p played an important role in the maintenance of malignant phenotypes of OC cells.

### CAV1 was an authentic target of miR-96-5p in OC cells

**Fig. 4:**
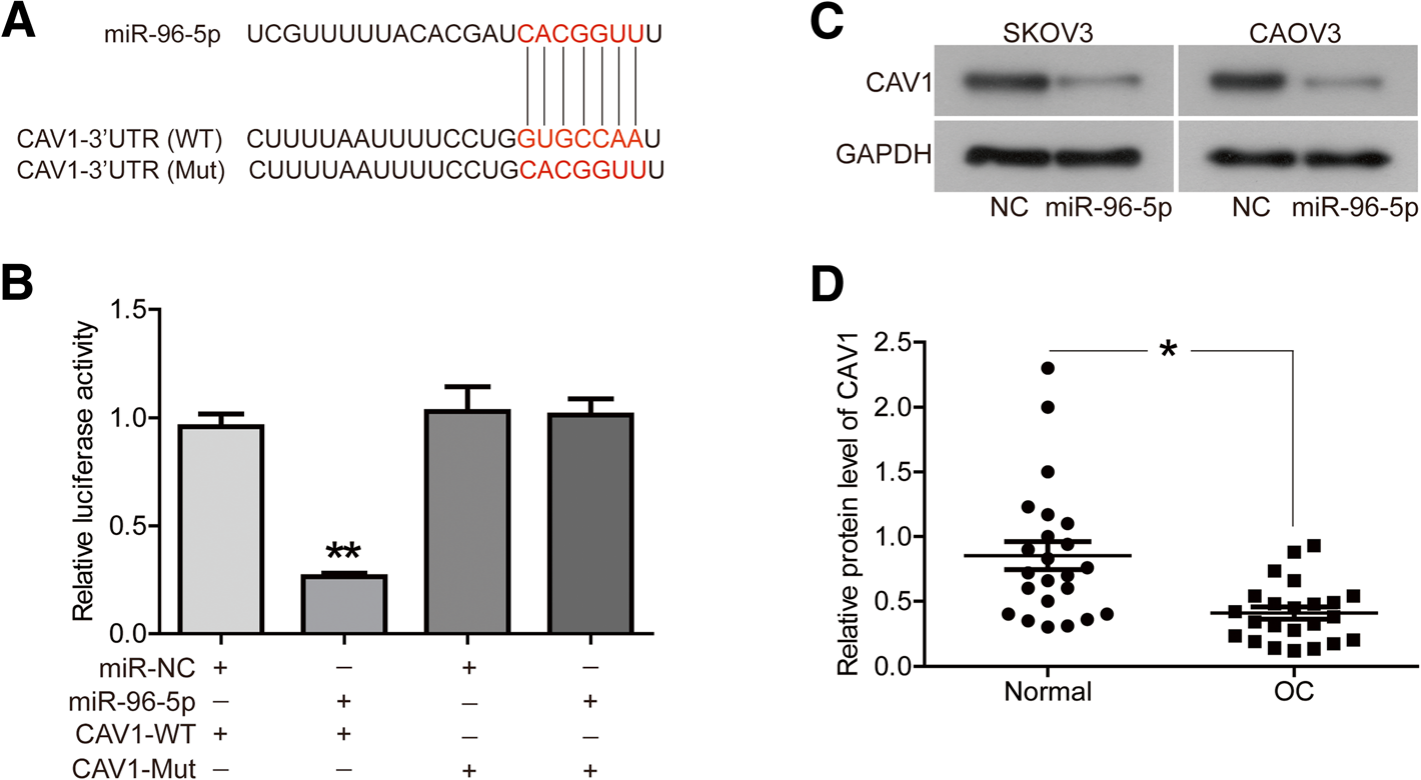
CAV1 was an authentic target of miR-96-5p in OC cells. **a** the sequences of miR-96-5p, CAV1 3′-UTR (WT) and mutant 3′-UTR (Mut). **b** the expression levels of luciferase of SKOV3 cells transfected with wild-type (WT) or mutated (Mut) CAV1 reporters plus miR-96-5p mimic or miR-NC were determined. **c** the protein expression of CAV1 were detected by western blot. **d** the protein expression of CAV1 in OC tissues and adjacent normal ovarian tissues (*n* = 23) were evaluated by western blot. *P < 0.05, **P < 0.01

Further, we identified a target gene of miR-96-5p on OC cells. The potential miR-96-5p targets were predicted using the TargetScanHuman (http://www.targetscan.org/). The prediction showed that CAV1 mRNA contained a potential miR-96-5p seed sequence within 3′ UTR (Fig. 4a). Furthermore, luciferase activity assays proved the direct relationship between CAV1 and miR-96-5p. As shown in Fig. 4b, co-transfection of miR-96-5p mimics and CAV1-WT led to the inhibition of luciferase activity (*P* < 0.01), whereas the cells transfected with CAV1-Mut did not exhibit decreased luciferase activity with or without the presence of miR-96-5p mimics. In addition, the overexpression of miR-96-5p significantly inhibited the protein expression of CAV1 in both cells (Fig. 4c). We also found the significantly low expression of CAV1 in same OC tissues described above, compared to normal ovarian tissues (Fig. 4d, *P* < 0.05).

### The overexpression of CAV1 abrogated the effect of miR-96-5p on OC cells

**Fig. 5:**
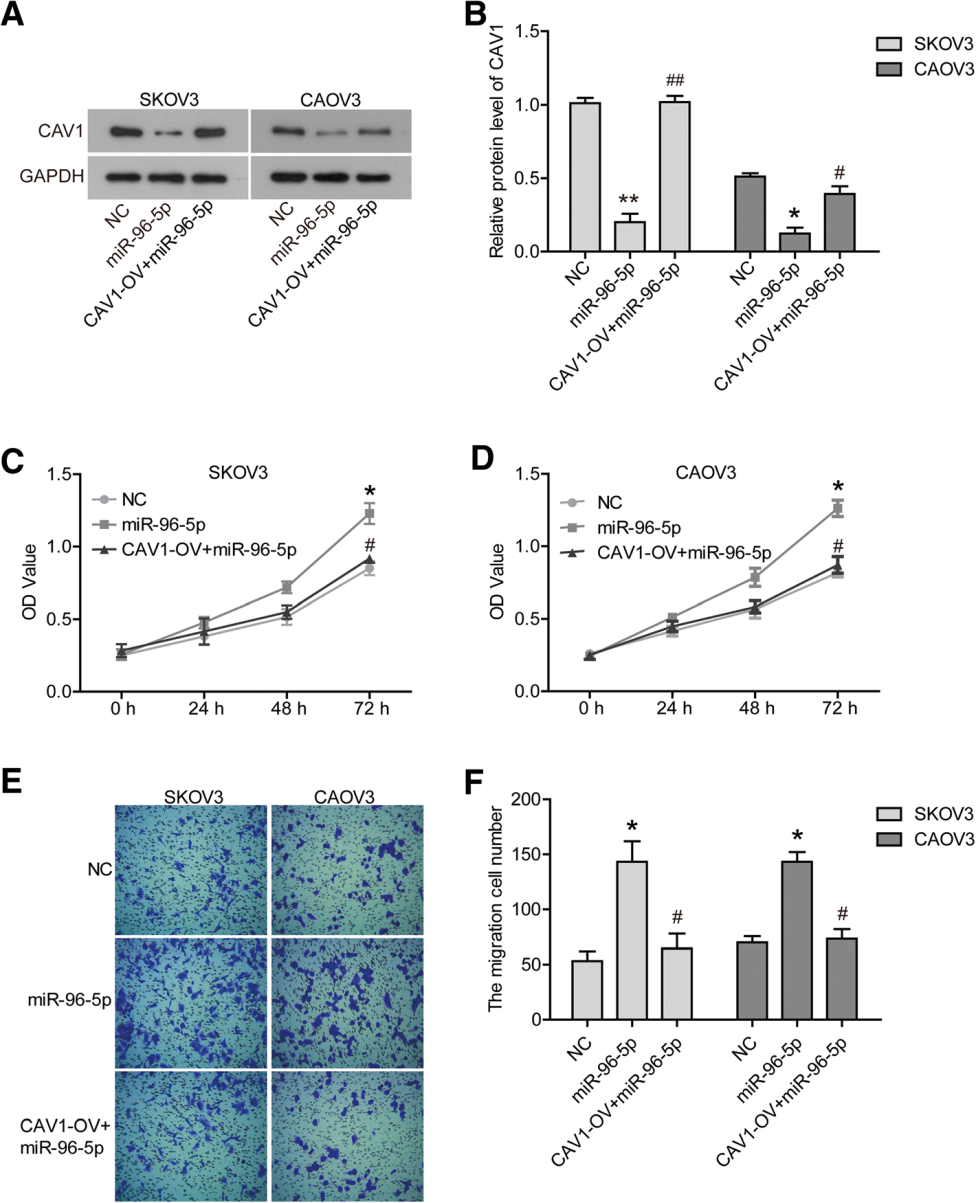
The overexpression of CAV1 abrogated the effect of miR-96-5p on OC cells. **a/b** the protein expression of CAV1 were detected by western blot. **c/d** the proliferation of SKOV3 and CAOV3 cells were determined via CCK8 assay. **e/f** the migration of SKOV3 and CAOV3 cells were determined by transwell assay. *P < 0.05 compared with NC. ^#^*P* < 0.05 compared with miR-96-5p. NC, negative control; miR-96-5p, miR-96-5p mimics transfected cells; CAV1-OV + miR-96-5p, CAV1 and miR-96-5p co-transfected cells

In addition, we tried to reveal if CAV1 was responsible for the effect of miR-96-5p on OC cells. A pcDNA3.1-CAV1 vector was transfected into cells to up-regulate the expression of CAV1 which was inhibited by miR-96-5p mimics (Fig. 5a/b, *P* < 0.05). Furthermore, CCK8 assays showed that CAV1 overexpression abrogated the promotion of miR-96-5p on the cells proliferation (Fig. 5c/d, *P* < 0.05). Transwell assays also demonstrated that the migration ability of cells was partially restrained by CAV1 overexpression under the existence of miR-96-5p (Fig. 5e/f, *P* < 0.05).

### AKT signaling pathway involved in the effect of miR-96-5p/CAV1 on OC cells

**Fig. 6:**
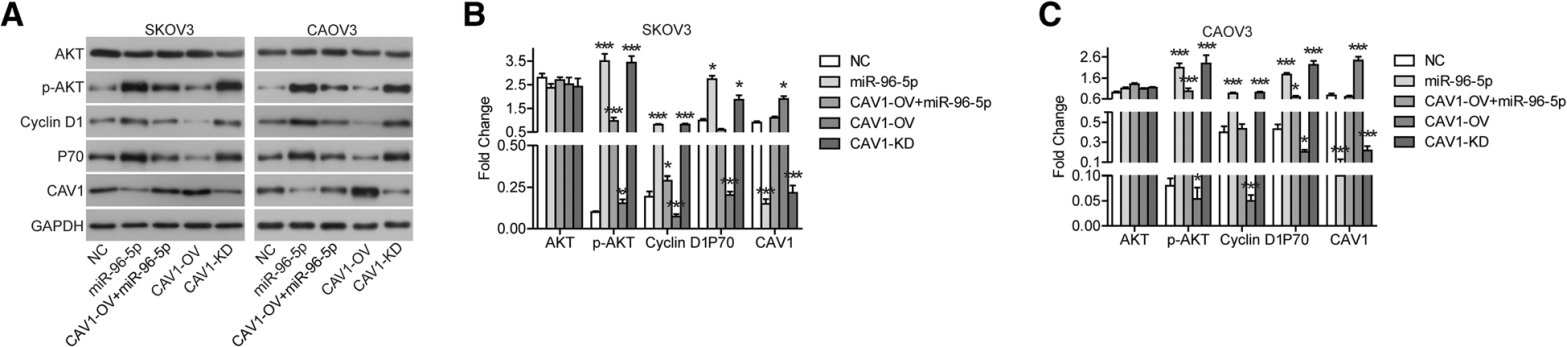
AKT signaling pathway involved in the effect of miR-96-5p/CAV1 on OC cells. **a-c** the protein expression were detected by western blot. *P < 0.05, **P < 0.01 compared with NC. NC, negative control; miR-96-5p, miR-96-5p mimics transfected cells; CAV1-OV + miR-96-5p, CAV1 and miR-96-5p co-transfected cells; CAV1-OV, CAV1 overexpression; CAV1-KD, CAV1 knockdown

Finally, we found that AKT signaling pathway involved in the effect of miR-96-5p/CAV1 on OC cells. As shown in Fig. 6, miR-96-5p mimics stimulated the phosphorylation of AKT, while had not effect on the protein expression of AKT. The expression of down-stream proteins, including Cyclin D1 and P70, was also up-regulated after transfection with miR-96-5p mimics. In the meantime, overexpression of CAV1 restrained the phosphorylation or expression of these proteins. Furthermore, wo examined the effects of CAV1 function obtaining or lossing on the AKT signaling pathway. Over-expression of CAV1 significantly inhibited phosphorylation of AKT and expression of Cyclin D1 and P70 (Fig. 6). The effect of CAV1 knockdown is exactly the opposite, but it is basically consistent with the effect of miR-96-5p overexpression (Fig. 6).

## Discussion

Firstly, in this study, we found that the abundance of miR-96-5p was significantly elevated in OC tissues and OC cell lines compared to normal ovarian tissues and epithelial cell line. In fact, miR-96-5p has been identified as related to various types of cancers. Similarly, miR-96-5p was also up-regulated in the serum samples from OC patients, compared to health participants. In addition, we found a positive correlation of miR-96-5p between OC tissues and serum samples. miR-96-5pIn fact, miR-96-5p has shown increased levels in serum samples from patients with breast cancer [8], prostate cancer [26], hepatocellular carcinoma [27], adrenocortical and adrenal medullary tumors [11] compared to healthy controls, and its level is closely related to tumor size, malignancy and metastasis. Our study revealed for the first time the expression pattern of miR-96-5p in OC tissues and cells, and in serum samples from OC patients, which were consistent with its expression patterns in other types of tumors.

Secondly, we found that overexpression of miR-96-5p promoted cell proliferation and migration of OC cells. MiR-96-5p seems to have special significance with the proliferation and motility of tumor cells. For example, overexpression of miR-96-5p promotes the proliferation of gastric cancer cell line (SGC7901), glioma cell lines (U87, U251) [28, 29], cervical cancer cell line (HeLa) [30], esophageal cancer cell lines (TE-1, Eca-109) and thyroid cancer cell lines (TPC1, K1) [9], while overexpression of miR-96-5p enhances invasion and migration of non-small cell lung cancer cell line (A549) [31] and colorectal cancer cell line (HCT116). And, miR-96-5p is also beneficial for proliferation and migration of pancreatic cancer (AsPC-1) and hepatocellular cancer cell lines (HepG2, HuH7, SK-Hep1, Hep3B) [27, 32]. Our results suggested that the effects of miR-96-5p on the proliferation and migration of OC cells were consistent with its effects on other types of tumor cells.

Further, we identified a new target of miR-96-5p, CAV1, on OC cells. CAV1 mRNA contained a potential miR-96-5p seed sequence within 3′ UTR. Luciferase activity assays also proved the direct relationship between CAV1 and miR-96-5p. In addition, overexpression of miR-96-5p significantly inhibited the protein expression of CAV1. These evidences did prove that CAV1 was the target of miR-96-5p. At present, 14 direct targets of miR-96-5p, including FOXO1, have been discovered [10], and we identified a new target in this study. Importantly, CAV1 was found to be low expression in the OC tissues. In addition, we found that overexpression of CAV1 abrogated the promotion of miR-96-5p on the SKOV3 cells proliferation and migration. Our results were consistent with the results of Prinetti et.al, that silencing of CAV1 strongly increased cell motility of OC cell line A2780 [20].

Finally, we found that AKT signaling pathway involved in the effects of miR-96-5p/CAV1 on OC cells. MiR-96-5p mimics stimulated the phosphorylation of AKT and the expression of Cyclin D1 and P70, while overexpression of CAV1 restrained the phosphorylation or expression of these proteins. AKT pathway is generally activated in OC cells [33]. Over-expression of CAV1 significantly inhibited phosphorylation of AKT and expression of Cyclin D1 and P70. The effect of CAV1 knockdown is exactly the opposite, but it is basically consistent with the effect of miR-96-5p overexpression. These results indicated that CAV1 was at least partially related to the regulation of AKT signaling pathway by miR-96-5p. Zou et.al also find that knockdown of CAV1 significantly down-regulates the protein expression of p-AKT in cisplatin-resistant OC cells (SKOV3/DDP and A2780/DDP cell lines) [21]. In addition, Sanan et.al believe that transient CAV1 transfection does not alter the activation of AKT in SKOV3 cells, and CAV1 can bind to Cyclin D1 promoter to influence its expression [34]. However, our current findings were not certain whether CAV1 can directly affect the expression of Cyclin D1 without affecting the activation of AKT.

## Conclusion

In conclusion, miR-96-5p was shown to be associated with OC and promote the proliferation and migration of OC cells by targeting CAV1. This finding implied that miR-96-5p was a potential therapeutic target for OC treatment.

## Methods

### Tissue samples

23 cases of primary epithelial ovarian cancer tissues and 23 cases of their corresponding adjacent normal ovarian tissues (located > 3 cm from the tumor) were collected from the OC patients receiving surgical resection at the Jinan Maternal & Children Health Care Hospital between June 2017 and November 2017. All patients’ written informed consent was obtained following the procedures approved by Jinan Maternal & Children Health Care Hospital. None of the patients had received any other treatment before surgery resection, including radiotherapy and chemotherapy.

### Serum collection

Serum samples were collected from 23 OC patients mentioned above and 23 health participants. Blood were collected from vena and transferred into serum collection tubes. They were centrifuged at 3000 g for 10 min at 4 °C. Before 1 h coagulation at room temperature, the serum samples were transferred into new tubes and stored at − 80 °C for further analysis.

### Cell culture and transfection

Human ovarian cancer cell lines (A2780, Hey, 3AO, OVCAR3, CAOV3 and SKOV3) and an immortalized normal human fallopain tube epithelial cell line (FTE187) were obtained from Cell Bank of Chinese Academy of Sciences (Shanghai, China). Cells lines were propagated in RPMI-1640 medium (Thermo Scientific Dionex, Shanghai, China) supplemented with 10% fetal bovine serum (FBS) (Invitrogen, Carlsbad, CA, USA), 100 units/ml penicillin, and 100 μg/ml streptomycin at 37 °C, in a 5% CO_2_.

Once cells were confluent between 60 and 70%, transfection could be performed immediately. MiR-96-5p mimics and CAV1 cDNA were transfected into cells with Lipofectamine 2000 Reagent (Invitrogen, San Diego, CA, USA) with manufacturer’s instructions. MiR-96-5p mimics and siRNA-CAV1 (CAV1-KD) were purchased from RiboBio (Guangzhou, China). CAV1-NC and CAV1 cDNA were synthesized by Sigma-Aldrich and GeneChem (Shanghai, China). CAV1-NC and CAV1 cDNA sequences were cloned into pCDNA3.1 vector (Invitrogen, Shanghai, China).

### Quantitative real-time PCR (qRT-PCR)

Total RNA was harvested from OC and normal ovarian tissues, serum samples and cultured cell using TRIzol (Invitrogen, San Diego, CA, USA), and 5 μg of RNA was synthesized into cDNA using a Reverse Transcription Kit (Takara Bio, Dalian, China). The quantitative PCR was performed in a Bio-Rad CFX96 real-time PCR system (Bio-Rad, CA) with a SYBR Green One (Takara Bio, Dalian, China). The data were analyzed with 2^-∆∆Ct^ method. GAPDH and U6 served as endogenous references for CAV1 and miR-96-5p, respectively.

### CCK8 assay

At 12 h after transfection, 5 × 10^3^ cells (100 μL) were planted into each well of 96-well plates. At the indicated time points (24, 48 and 72 h), the cultured medium was removed and changed to fresh medium containing 10 μl CCK8 solution. Then cells were maintained in incubator for 4 h. The absorbance values were read with a microplate reader set at 450 nm.

### Colony formation assay

At 48 h post transfection, 200 cells were routine cultured in 6-well plate for 14 days. New medium were replaced every 3 days. When colonies were obviously visible, they were fixed with 95% ethanol and stained with 0.1% crystal violet for counting under a microscope.

### Transwell assay

Transwell assay was performed with 8 μm chambers. Cells were dissolved in the RPMI-1640 medium without FBS. 100 μL of cells (1 × 10^6^ cells/mL) was plated into upper chambers and 500 μL RPMI-1640 medium supplemented with 10% FBS was added as chemotactic factor into lower chambers of 24-well plates. 24 h later, non-migrating cells were removed while migrating cells were fixed by methanol and then dyed with crystal violet. They were observed and counted under a microscope.

### Flow cytometry for cell cycle detection

After 48 h post transfection, cells were trypsinized (without EDTA), and resuspended in cold PBS. Then cells were fixed by cold 70% ethanol at − 20 °C for 24 h. Fixed cells were incubated with RNase A for 30 min, and stained with propidium oxide for another 30 min in dark by Cell Cycle Analysis Kit (Beyotime Biotechnology, Beijing, China). The cell cycle progression were immediately detected using a flow cytometer, and analyzed using FCS Express 4 software (De NovoSoftware, Los Angeles, CA).

### Dual luciferase reporter assay

The DNA fragment of the specifical sites in the wild type (CAV1-WT) and mutated (CAV1-Mut) CAV1 3′ UTR was synthesized in vitro and cloned into psiCHECK-2 Vector (Promega, Madison, WI, USA). Then the vectors were co-transfected with miR-96-5p mimics or miR-NC for 48 h into the SKOV3 cells. The luciferase assays were performed by Luciferase Assay Kit (Promega, WY).

### Western blot

Proteins were extracted from cells using ice-cold RIPA buffer (Beyotime, Shanghai, China) supplemented with protease inhibitors and phosphase inhibitors. The protein concentrations were measured by BCA method. Then 20 μg of protein were separated by sodium dodecyl sulfate polyacrylamide gel electrophoresis (SDS-PAGE) and transferred onto a 0.45 μm PVDF membrane. The membrane was blocked with 5% skim milk for 1 h and incubated with primary antibodies overnight. After washing with PBS, blots were incubated with secondary antibodies. Images were acquired by enhanced chemiluminescence. GAPDH antibody was used as a control. Results were treated with gray analysis by Gel-Pro Analyzer (United States Biochemical, Cleveland, OH). The semi-quantitative analysis was performed according to the relative expression of objective protein and GAPDH.

The primary antibodies used in this study were as follow: anti-CAV1 (1:1000, Rabbit polyclonal antibody, ab18199, Abcam, Cambridge, MA, USA); anti-AKT (1:500, Mouse monoclonal antibody, #9272, Cell Signaling Technology, Beverly, MA, USA); anti-p-AKT (1:1000, Mouse monoclonal antibody, Ser473, 66,444–1-Ig, Proteintech, Manchester, UK); anti-Cyclin D1 (1:1000, Rabbit monoclonal antibody, ab16663, Abcam); anti-P70 (1:1000, Rabbit monoclonal antibody, ab184551, Abcam); anti-GAPDH (1:500, Mouse monoclonal antibody, ab8245, Abcam).

### Statistical analysis

With SPSS 21.0 (SPSS Inc., Chicago, IL, USA) and GraphPad 7.0 (GraphPad Software, La Jolla, CA, USA), all statistical analyses were presented as mean ± standard deviation (SD). Unpaired student’s t-test was used for comparison in two parts. One-factor analysis of variance (ANOVA) test was used for statistical analysis in three or most parts. A *P* < 0.05 indicated a statistical significance. Each experiment included triplicate measurements for each condition tested.

## Data Availability

The datasets used and/or analysed during the current study are available from the corresponding author on reasonable request.

## References

[1] Mandilaras V, Vernon M, Meryetfiguière M, Karakasis K, Lambert B, Poulain L (2017). Updates and current challenges in microRNA research for personalized medicine in ovarian cancer. Expert Opin Biol Ther.

[2] Lewis BP, Burge CB, Bartel DP (2005). Conserved seed pairing, often flanked by adenosines, indicates that thousands of human genes are microRNA targets. Cell..

[3] Gabra MM, Salmena L. microRNAs and acute myeloid leukemia Chemoresistance: a mechanistic overview. Front Oncol. 2017;7:255. 10.3389/fonc.2017.00255 PMC5674931 29164055

[4] Chen PS, Su JL, Hung MC (2012). Dysregulation of microRNAs in cancer. J Biomed Sci.

[5] Nugent M (2011). MicroRNAs in colorectal cancer: function, dysregulation and potential as novel biomarkers. Eur J Surg Oncol.

[6] Nowroji K, Soundararajan V, Subramanion Lachumy J, Chern Ein O, Yeng C, Jagat Rakesh K (2014). MicroRNAs: biogenesis, roles for carcinogenesis and as potential biomarkers for cancer diagnosis and prognosis. Asian Pac J Cancer Prev.

[7] Galasso M, Sandhu SK, Volinia S (2012). MicroRNA expression signatures in solid malignancies. Cancer J.

[8] Xie W, Sun F, Chen L, Cao X (2018). miR-96 promotes breast cancer metastasis by suppressing MTSS1. Oncol Lett.

[9] Hong-Ming S, Yi L, Deng-Feng L, Chuan-Kui W, Kai-Yao H, Jia-Lu S (2015). MicroRNA-96 plays an oncogenic role by targeting FOXO1 and regulating AKT/FOXO1/Bim pathway in papillary thyroid carcinoma cells. Int J Clin Exp Pathol.

[10] Wu Z, Liu K, Wang Y, Xu Z, Meng J, Gu S (2015). Upregulation of microRNA-96 and its oncogenic functions by targeting CDKN1A in bladder cancer. Cancer Cell Int.

[11] Xu Y, Wang Z, Bi Y, Duan Z, Yue X (2018). Correlation between CT features of adrenocortical and adrenal medullary tumors and expression of miR-96 in serum. Oncol Lett.

[12] Qin Y, Pei Y, Xin Z, Xie B. microRNA-96 acts as a tumor suppressor gene in human osteosarcoma via target regulation of EZRIN. Life Sci. 2018; S0024320518301930. 10.1016/j.lfs.2018.04.012 29656060

[13] Alex WC, Robert H, William S, Michael PL (2004). Role of caveolae and caveolins in health and disease. Physiol Rev.

[14] Chidlow JH, Sessa WC (2010). Caveolae, caveolins, and cavins: complex control of cellular signalling and inflammation. Cardiovasc Res.

[15] Galbiati F, Razani B, Lisanti MP (2001). Caveolae and caveolin-3 in muscular dystrophy. Trends Mol Med.

[16] Galbiati F, Razani B, Lisanti MP (2001). Emerging themes in lipid rafts and caveolae. Cell..

[17] Watanabe M, Yang G, Cao G, Tahir SA, Naruishi K, Tabata K (2009). Functional analysis of secreted caveolin-1 in mouse models of prostate cancer progression. Mol Cancer Res.

[18] Anbarasu K, Arunkumar K, Mohammed A, Shyama S, Devaraj H (2014). Caveolin-1 promotes gastric cancer progression by up-regulating epithelial to mesenchymal transition by crosstalk of signalling mechanisms under hypoxic condition. Eur J Cancer.

[19] Gustavo A, Matteo M, Anna F, Sungyong Y, Rile L, Fabiana R (2013). Loss of caveolin-1 in prostate cancer stroma correlates with reduced relapse-free survival and is functionally relevant to tumour progression. J Pathol.

[20] Alessandro P, Ting C, Giuditta I, Simona P, Massimo A, Nicoletta G (2011). A glycosphingolipid/caveolin-1 signaling complex inhibits motility of human ovarian carcinoma cells. J Biol Chem.

[21] Zou W, Ma X, Hua W, Chen B, Cai G (2015). Caveolin-1 mediates chemoresistance in cisplatin-resistant ovarian cancer cells by targeting apoptosis through the Notch-1/Akt/NF-kappaB pathway. Oncol Rep.

[22] Jamil D, Qing X, Raia D, Martin C (2002). TEL/platelet-derived growth factor receptor beta activates phosphatidylinositol 3 (PI3) kinase and requires PI3 kinase to regulate the cell cycle. Blood..

[23] Nirmalya D, Nandini GC, Kasinath BS, Goutam Ghosh C (2012). TGFβ-stimulated microRNA-21 utilizes PTEN to orchestrate AKT/mTORC1 signaling for mesangial cell hypertrophy and matrix expansion. PLoS One.

[24] Ataie-Kachoie P, Pourgholami MH, Bahrami-B F, Badar S, Morris DL (2015). Minocycline attenuates hypoxia-inducible factor-1α expression correlated with modulation of p53 and AKT/mTOR/p70S6K/4E-BP1 pathway in ovarian cancer: in vitro and in vivo studies. Am J Cancer Res.

[25] Chunliu M, Juan M, Hui S, Jing L, Fei W, Jung Joon L (2014). 4′,6-dihydroxy-4-methoxyisoaurone inhibits the HIF-1α pathway through inhibition of Akt/mTOR/p70S6K/4E-BP1 phosphorylation. J Pharmacol Sci.

[26] Xu L, Zhong J, Guo B, Qi Z, Hang L, Wen N (2016). miR-96 promotes the growth of prostate carcinoma cells by suppressing MTSS1. Tumor Biol.

[27] Li Z, Wang Y (2018). miR-96 targets SOX6 and promotes proliferation, migration, and invasion of hepatocellular carcinoma. Biochem Cell Biol.

[28] Zhiyong Y, Jianpeng W, Chao W, Yingbing J, Weiguo Q, Shusheng C (2014). miR-96/HBP1/Wnt/β-catenin regulatory circuitry promotes glioma growth. FEBS Lett.

[29] Ma QQ, Huang JT, Xiong YG, Yang XY, Han R, Zhu WW (2017). MicroRNA-96 regulates apoptosis by targeting PDCD4 in human glioma cells. Technol Cancer Res Treat.

[30] Ma X, Shi W, Peng L, Qin X, Hui Y (2018). MiR-96 enhances cellular proliferation and tumorigenicity of human cervical carcinoma cells through PTPN9. Saudi J Biol Sci.

[31] Fei X, Zhang J, Zhao Y, Sun M, Zhao H, Li S (2018). miR-96 promotes invasion and metastasis by targeting GPC3 in non-small cell lung cancer cells. Oncol Lett.

[32] Wang TH, Yeh CT, Ho JY, Ng KF, Chen TC (2016). OncomiR miR-96 and miR-182 promote cell proliferation and invasion through targeting ephrinA5 in hepatocellular carcinoma. Mol Carcinog.

[33] Linnerth-Petrik NM, Santry LA, Moorehead R, Jücker M, Wootton SK, Petrik J (2016). Akt isoform specific effects in ovarian cancer progression. Oncotarget..

[34] Sanna E, Miotti S, Mazzi M, Santis GD, Canevari S, Tomassetti A (2007). Binding of nuclear caveolin-1 to promoter elements of growth-associated genes in ovarian carcinoma cells. Exp Cell Res.

